# The Unseen Picture: Issues with Health Care, Discrimination, Police and Safety, and Housing Experienced by Native American Populations in Rural America

**DOI:** 10.1111/jrh.12517

**Published:** 2020-10-06

**Authors:** Mary T.G. Findling, Robert J. Blendon, John M. Benson, Carolyn Miller

**Affiliations:** ^1^ Department of Health Policy & Management Harvard T.H. Chan School of Public Health Boston Massachusetts; ^2^ Research, Evaluation, and Learning Unit Robert Wood Johnson Foundation Princeton New Jersey

**Keywords:** American Indian/Alaska Natives, health care, Native Americans, rural health, social determinants of health

## Abstract

**Purpose:**

Despite increased national attention to improving rural health, rural Native American populations face unique problems that are often unseen in aggregate research on the rural United States. The objective of this study was to examine rural Native Americans’ experiences with serious problems across domains important to health, using rural Whites as a comparison group.

**Methods:**

Using 2 probability‐based national telephone surveys (2017 and 2019), we examined rural Native American adults’ reported problems in health care, discrimination, police and safety, and housing. We then compared Native American‐White differences in reported problems across domains.

**Findings:**

Among rural Native American adults, 33% reported recent problems accessing health care when they needed it, 28% reported they or family members recently experienced major problems paying for medical bills, and 28% reported recent problems with health care quality. Several Native American‐White differences were reported, including experiencing racial violence (34% vs 5%, *P* < .001), discrimination in health care (19% vs 3%, *P* = .003), unfair police treatment (27% vs 5%, *P* = .002), and major housing problems (48% vs 26%, *P* < .001).

**Conclusions:**

Rural Native American adults report ongoing and widespread problems with health care, discrimination, the police, safety, and housing. These findings support many national policy recommendations to improve federal funding and oversight for programs serving Native American populations living in rural areas.

Despite recent increases in national attention to improving rural health,^1^, ^2^, ^3^ Native American populations living on tribal lands and in other rural areas face unique problems often unseen in the larger picture of research on rural America. In particular, survey research has been limited in its ability to include representative samples of rural Native Americans because they are hard to reach,^4^, ^5^, ^6^ though recent news highlights several issues they face. For example, *Wall Street Journal* investigations documented major shortfalls in health care provided to rural Native American populations through the Indian Health Service, including calls from the Department of Health and Human Services’ Office of Inspector General to improve problems with health care funding, quality, and oversight.^7^, ^8^ In addition, the Government Accountability Office, Department of Justice, and US Commission on Civil Rights have repeatedly requested improved federal funding and oversight for Native American populations living on tribal lands and in other rural areas.^9^, ^10^, ^11^, ^12^, ^13^

Rural Native American populations face distinct and exacerbated health disparities compared to their White counterparts.^2^, ^5^, ^14^, ^15^, ^16^ Though precise estimates vary depending on both the definition of rural and the inclusion of race as American Indian/Alaska Native alone or multiracial, a significant share of Native Americans live in rural America, both within and outside of tribal lands.^17^, ^18^ However, because they comprise a low percentage of the total rural population, rural Native Americans are not typically well represented in national surveys.^4^, ^5^, ^6^

The Centers for Disease Control and Prevention gathers data on the rural Native American populations’ basic health status and behavioral indicators,^5^ but research gaps remain examining their reported life experiences apart from the general rural population. To our knowledge, no prior probability‐based studies using national samples have recently examined rural Native Americans’ personally reported experiences with serious health care, discrimination, police and safety, or housing problems. We conducted 2 polls in 2017 and 2019 to examine their experiences, using rural Whites as a comparison group.

## Methods

### Study Design and Sample

Data used in this study were obtained from 2 original, nationally representative, probability‐based cell and landline telephone polls of US adults. Rural was defined as geographic areas outside of metropolitan statistical areas. The first poll was conducted from January 26 to April 9, 2017, about experiences of discrimination against several minority groups in America. The second poll was conducted from January 31 to March 2, 2019, on economic‐ and health‐related experiences facing adults in rural America. Both surveys were jointly designed by Harvard T.H. Chan School of Public Health, the Robert Wood Johnson Foundation, and National Public Radio and were fielded by SSRS, an independent survey research firm. Harvard researchers were not directly involved in data collection and de‐identified datasets were used for analysis, thus the Harvard T.H. Chan School of Public Health Office of Human Research Administration classified these surveys as “not human subjects research.”

The final analytic samples included 317 Native American adults and 1,066 White adults aged 18 years and above living in the rural United States. Native Americans were oversampled in both surveys to obtain adequate sample sizes for the analysis. For race, respondents self‐identified as Native American, American Indian, or Alaska Native. Multiracial respondents were asked which race they identified with most and were included in the sample if they identified mostly as Native American, and “Native American” was used in follow‐up questions.

Survey completion rates were 74% (survey 1) and 76% (survey 2) among respondents who answered initial demographic screening questions. Overall response rates were 10% (survey 1) and 8% (survey 2), calculated based on the American Association for Public Opinion Research's (AAPOR) RR3 formula.^19^ While these response rates are not ideal, they are consistent with response rates of telephone polls by prominent survey organizations.^20^

### Survey Instrument

Questions were developed following AAPOR best practices, after conducting a review of the existing survey data. Questionnaires were reviewed by external experts for bias, balance, and comprehension and were pretested among a subset of respondents before being conducted among the full samples. We analyzed 16 questions. Some questions (eg, discrimination) were only asked among a randomized half‐sample of respondents to limit the burden on their time. For sensitive topics such as harassment, we used a validated method^21^ to ask whether some experiences happened to the respondent or their family members. Question wording is available in online Appendix A.

### Statistical Analyses

After calculating descriptive statistics, we calculated the percentages of adults reporting their experiences for each question using survey weights. We used pairwise *t* tests of differences in proportions to make uncontrolled comparisons between rural Native American and White respondents. We only report results with at least 10 percentage points’ difference as robust enough to have statistical and practical implications, with statistical significance at *P* < .05.

To compensate for nonresponse bias and variations in the probability of selection, data were weighted by household size and composition, cell phone and landline use, and demographics using US Census data on gender, age, education, race/ethnicity, and region to reflect the true population distribution of Native American and White adults in the rural United States. We also used random‐digit dialing and random selection of respondents within households to attain a representative sample. Analyses were conducted using STATA version 15.0 (StataCorp LLC, College Station, TX).

## Results

The characteristics of rural adults from 2 surveys included in this study are presented in Table 1. Most rural Native Americans did not have a college degree, and a majority lived in households earning < $50,000 annually. Among rural Whites, most did not have a college degree, while approximately half lived in households earning < $50,000 annually.

**Table 1:** Characteristics of the Study Samples of Rural Native American and White Adults^a^

	Survey 1		Survey 2	
	Rural Native Americans (N = 178)^b^	Rural Whites (N = 174)	Rural Native Americans (N = 139)	Rural Whites (N = 892)
	Weighted percentage of respondents^c^			
Gender				
Male	44	48	48	49
Female	56	52	52	51
Age				
18–29 y	20	21	15	18
30–64 y	61	49	60	54
65+ y	18	30	25	27
Education				
No college degree^d^	85	80	89	79
College degree or more	14	20	11	20
Household income				
<$25,000	46	32	49	27
$25,000–<$50,000	26	21	25	21
$50,000–<$75,000	8	15	8	17
$75,000+	19	26	12	24
US region of residence^e^				
Northeast	4	10	2	13
Midwest	19	28	25	36
South	38	43	44	37
West	39	18	28	15

^a^ Native American and White adults ages 18+.

^b^ The sample size shown reflects the total number of respondents in each category.

^c^ Percent of US population estimated with survey weights to adjust for unequal probability of sampling; may not add up to 100% due to rounding and do not know/refused responses that are included in the total n but not reported.

^d^ Includes those with some college experience (including business, technical, or vocational school after high school) but no college degree, as well as those with a high school degree or General Educational Development certificate or less.

^e^ Regions defined by US Census Bureau 4‐region definition.

Table 2 shows reported experiences with health care, discrimination, police and safety, and housing problems. For health care, 33% of rural Native Americans reported problems accessing health care when they needed it in the past few years, while 28% reported they or family members experienced major problems paying for medical bills in the past few years. In addition, 28% of rural Native Americans reported recent problems with health care quality. There were no statistically significant differences between rural Native Americans and Whites on any health care measures.

**Table 2:** Differences in Reported Experiences with Health Care, Discrimination, Police and Safety, and Housing Issues between Rural Native Americans and Rural Whites

	N^a^	Rural Native Americans weighted %	Rural Whites weighted %	*P* value for difference
*Health care*				
Experienced problems with health care access	1,031	33	27	.284
Experienced major problems paying for medical bills	1,031	28	19	.091
Experienced problems with health care quality^b^	515	28	29	.824
*Discrimination*				
Experienced discrimination when trying to rent a room/apartment or buy a house^b,c^	141	24	4	.015*
Experienced discrimination in police interactions^b^	169	22	7	.024*
Avoided calling the police because of concerns of discrimination^b^	169	21	0	.004*
Experienced discrimination when going to a doctor or health clinic^b^	183	19	3	.003*
Avoided doctor or health care because of concerns of discrimination/poor treatment^b^	183	14	4	.046*
*Police and Safety*				
Been threatened or harassed^b,d^	169	37	10	<.001*
Experienced violence^b,d^	169	34	5	<.001*
Rated local community as unsafe from crime^b^	515	27	10	.016*
Unfairly stopped or treated by the police^b,d^	169	27	5	.002*
Unfairly treated by the courts^b,d^	169	23	8	.039*
*Housing*				
Experienced any major housing problems	1,031	48	26	<.001*
Experienced major problems paying for housing	1,031	20	9	.015*
Reported homelessness is a major problem in the local community^b^	520	19	16	.564

* Rural Native American adults significantly different from rural White adults at *P* < .05.

^a^ Authors’ analysis of 2 surveys of 317 Native American and 1,066 non‐Hispanic White adults living in the rural United States (total N across both surveys, all analyses use weighted data). Do not know/refused responses included in the total N for each question. Full question wording is available in online Appendix A.

^b^ Question only asked among a half‐sample of respondents to limit the time burden.

^c^ Housing question only asked among respondents who have ever tried to rent a room or apartment, or to apply for a mortgage or buy a home.

^d^ Questions asked if events have happened to you or a family member because you or they are [Native American or White].

On the topic of discrimination, more than 1 in 5 rural Native Americans reported experiencing racial discrimination in several areas, including when trying to find housing (24%), discrimination in police interactions (22%), and avoiding calling the police due to concerns about discrimination (21%). About 1 in 5 rural Native Americans (19%) reported experiencing racial discrimination when going to a doctor or health clinic, while 14% reported avoiding seeking needed health care due to fear of unfair treatment. Higher shares of rural Native Americans reported problems across all discrimination measures compared to rural Whites (Whites reporting housing discrimination: 4%, *P* = .015; discrimination in police interactions: 7%, *P* = .024; avoided calling police: 0%, *P* = .004; discrimination in health care: 3%, *P* = .003; avoided health care: 4%, *P* = .046).

When it comes to police and safety, 37% of rural Native Americans reported race‐related threats or harassment against themselves or family members and 34% reported racial violence against themselves or family members. More than 1 in 4 rural Native Americans (27%) rated their local community as unsafe from crime, 27% also reported they or Native American family members were unfairly stopped or treated by the police, and 23% reported they or family members were unfairly treated by the courts because of their race. Higher shares of rural Native Americans reported problems across all police and safety measures compared to rural Whites (Whites reporting racial threats/harassment: 10%, *P* ≤ .001; racial violence: 5%, *P* ≤ .001; community unsafe from crime: 10%, *P* = .016; unfair police treatment: 5%, *P* = .002; unfair treatment by courts: 8%, *P* = .039).

In housing, 48% of rural Native Americans reported any major housing problems while living in their current residence, including problems with drinking water safety, electricity, and mold or other environmental problems. Online Appendix B contains details on housing problems. Financially, 20% of rural Native Americans reported major problems paying for housing within the past few years, while 19% reported homelessness as a major problem in their local community. Compared to rural Whites, higher shares of rural Native Americans reported major housing problems (Whites: 26%, *P* < .001) and major problems paying for housing (Whites: 9%, *P* = .015). There was not a statistically significant difference between Native Americans and Whites for viewing homelessness as a problem.

## Discussion

This study provides a snapshot of rural Native American adults’ reported life experiences across areas that may drive their poor health outcomes observed in other studies,^5^, ^15^ with 4 important findings.

First, despite living in the post‐Affordable Care act era, at least one‐quarter of the rural Native American population reported facing serious problems with health care costs, access, and quality. Similar shares of rural Native American and White adults reported these health care problems, suggesting greater rural‐urban disparities in health care than racial disparities within rural areas. Several provisions in the Affordable Care Act have the potential to improve health care for both rural communities and Native American populations. However, in order to meaningfully improve health care access, affordability, and quality, policy makers should also address long‐standing underfunding of the Indian Health Service and other common barriers to care for rural Americans, including access to specialists.^12^, ^13^, ^22^, ^23^

Second, it is concerning that one‐fifth of the rural Native American population reported discrimination in clinical health care encounters and 1 in 7 avoided seeking health care due to anticipated discrimination. These results underscore significant policy opportunities identified elsewhere to eliminate discrimination and unfair treatment of Native Americans, in health care and beyond.^14^, ^24^

Third, rural Native American adults widely reported experiencing violence, harassment, discrimination, and unfair treatment by the police and courts, which is consistent with prior research findings without regard to rurality.^11^, ^12^, ^25^, ^26^, ^27^ While national attention to racism within policing has increased in recent years, it has largely centered on the experiences of Black Americans.^10^, ^28^ Additional, focused attention is needed to improve law enforcement and reduce discriminatory policing practices impacting Native American populations living in rural areas.^11^, ^12^, ^25^, ^26^

Fourth, nearly half of rural Native American adults reported at least one major problem while in their current residence, which is consistent with other research documenting housing shortages in Indian Country, and substandard housing where housing exists.^10^, ^12^ More research and funding are needed to support rural Native American populations’ unique housing problems, as distinct from rural communities generally.

### Limitations

Several limitations should be considered when interpreting our results. Although we oversampled rural Native American adults, both the sample size and some questions that were only asked of half the sample constrained our ability to examine heterogeneity within their diverse experiences across different geographies, cultures, heritage, traditions, and tribal affiliations. Future research should explore these differences, as well as protective factors that may improve experiences and health outcomes in areas we studied. Self‐reported data may introduce recall bias, and responses to sensitive topics may be underreported.^21^ We also were not able to compare rural Native Americans to their nonrural counterparts. Because the sample was not evenly distributed geographically across Native American and White populations, regional differences may have impacted reported experiences. Our low response rate is an important limitation, though prior studies suggest that low response rates do not bias results if respondents are representative of the study population.^20^, ^29^ If surveys with low response rates use probability‐based samples and are weighted using Census parameters, they are expected to yield accurate estimates in most cases.^20^, ^29^, ^30^, ^31^ However, selection bias may remain related to the measured experiences. Despite these limitations, this study allowed us to examine experiences of rural Native Americans, which is difficult in survey research due to sampling challenges.^4^, ^5^, ^6^

## Conclusions

Rural Native American populations report widespread serious problems with health care, discrimination, the police and safety, and housing, which are all likely to be exacerbated by the COVID‐19 pandemic.^32^ These problems are often unseen in the larger picture of research on rural America, as they differ from serious problems reported by rural Whites. Our findings support many national policy recommendations to increase federal funding and improve oversight for programs serving Native American populations living in rural areas, with goals of improving their health outcomes and reducing health disparities.^8^, ^9^, ^10^, ^11^, ^12^, ^13^, ^14^

## Supporting information

Supporting Information Available Online: Appendices A and B

## References

[1] Bolin JN , Bellamy GR , Ferdinand AO , et al. rural Healthy people 2020: new decade, same challenges. J Rural Health. 2015;31(3):326‐333. 25953431 10.1111/jrh.12116

[2] Meit M , Knudson A . Leveraging interest to decrease rural health disparities in the United States. Am J Public Health. 2017;107(10):1563‐1564. 28902537 10.2105/AJPH.2017.304025 PMC5607699

[3] U.S. Department of Agriculture . The White House Rural Forum. Press Release No. 0213.16. 2016. Available at: https://www.usda.gov/media/press-releases/2016/10/04/fact-sheet-white-house-rural-forum. Accessed September 3, 2020.

[4] Kalton G . “Chapter 19—Probability sampling methods for hard‐to‐sample populations.” In Tourangeau R , Johnson TP , Wolter KM , Bates N , eds. Hard‐to‐Survey Populations Cambridge, UK: Cambridge University Press; 2014:401‐423.

[5] James CV , Moonesinghe R , Wilson‐Frederick SM , Hall JE , Penman‐Aguilar A , Bouye K . Racial/ethnic disparities among rural adults—United States, 2012–2015. MMWR Surveill Summ. 2017;66(23):1‐9. 10.15585/mmwr.ss6623a1 PMC5829953 29145359

[6] Holm JE , Vogeltanz‐Holm N , Poltavski D , et al. Assessing health status, behavioral risks, and health disparities in American Indians living on the Northern Plains of the U.S. Public Health Rep. 2010;125(1):68‐78. 20402198 10.1177/003335491012500110 PMC2789818

[7] Wilde Mathews A , Weaver C . Six CEOs and no operating room: the impossible job of fixing the Indian Health Service. Wall Street Journal. 2019Available at: https://www.wsj.com/articles/six-ceos-and-no-operating-room-the-impossible-job-of-fixing-the-indian-health-service-11575993216. Accessed September 3, 2020.

[8] U.S. Department of Health and Human Services . Office of Inspector General. Organizational challenges to improving quality of care in Indian Health Service hospitals. OEI‐06‐16‐00390; 2019. Available at: https://oig.hhs.gov/oei/reports/oei-06-16-00390.pdf. Accessed September 3, 2020.

[9] Government Accountability Office . Testimony before the Committee on Indian Affairs, U.S. Senate. High risk—progress made but continued attention needed to address management weaknesses at federal agencies serving Indian tribes. 2019. Available at: https://www.gao.gov/assets/700/697490.pdf. Accessed September 3, 2020.

[10] Government Accountability Office . Native American housing—additional actions needed to better support tribal efforts. 2014. Available at: https://www.gao.gov/assets/670/662063.pdf. Accessed September 3, 2020.

[11] Wakeling S , Jorgensen M , Michaelson S , et al. Policing on American Indian reservations, a report to the National Institute of Justice. U.S. Department of Justice, Office of Justice Programs. 2001. Available at: https://www.ncjrs.gov/pdffiles1/nij/188095.pdf. Accessed September 3, 2020.

[12] U.S. Commission on Civil Rights, Office of Civil Rights Evaluation . A Quiet Crisis: Federal Funding and Unmet Needs in Indian Country. Washington, DC: U.S. Commission on Civil Rights; 2003. Available at: https://www.usccr.gov/pubs/na0703/na0204.pdf. Accessed September 3, 2020.

[13] U.S. Commission on Civil Rights . Briefing report—broken promises: continuing federal funding shortfall for Native Americans. 2018. Available at: https://www.usccr.gov/pubs/2018/12-20-Broken-Promises.pdf. Accessed September 3, 2020.

[14] Institute of Medicine . Unequal Treatment: Confronting Racial and Ethnic Disparities in Health Care. Washington, DC: The National Academies Press; 2003. 25032386

[15] Indian Health Service . Disparities. 2018. Available at: https://www.ihs.gov/newsroom/factsheets/disparities/. Accessed September 3, 2020.

[16] Bennett K , Olatosi B , Probst J . Health Disparities: A Rural–Urban Chartbook. Columbia, SC: South Carolina Rural Health Research Center; 2008.

[17] U.S. Census . The American Indian and Alaska Native population. 2010. Available at: https://www.census.gov/history/pdf/c2010br-10.pdf. Accessed September 3, 2020.

[18] U.S. Department of Health and Human Services , Office of Minority Health. Profile: American Indian/Alaska Native. 2018. Available at: https://minorityhealth.hhs.gov/omh/browse.aspx?lvl=3&26lvlid=62. Accessed September 3, 2020.

[19] American Association for Public Opinion Research . Standard definitions: final disposition case codes and outcome rates for surveys. 2016. Available at: https://www.aapor.org/AAPOR_Main/media/publications/Standard-Definitions20169theditionfinal.pdf. Accessed September 3, 2020.

[20] Keeter S , Hatley N , Kennedy C , et al. What Low Response Rates Mean for Telephone Surveys. Washington, DC: Pew Research Center. 2017. Available at: http://www.pewresearch.org/2017/05/15/what-low-response-rates-mean-for-telephone-surveys/. Accessed September 3, 2020.

[21] Tourangeau R , Yan T . Sensitive questions in surveys. Psychol Bull. 2007;33(5):859‐883. 10.1037/0033-2909.133.5.859 17723033

[22] Sequist TD , Cullen T , Bernard K , Shaykevich S , Orav EJ , Ayanian JZ . Trends in quality of care and barriers to improvement in the Indian Health Service. J Gen Intern Med. 2011;26(5):480‐486. 21132462 10.1007/s11606-010-1594-4 PMC3077488

[23] Sequist TD , Cullen T , Acton KJ . Indian Health Service innovations have helped reduce health disparities affecting American Indian and Alaska Native people. Health Affairs. 2011;10:1965‐1973. 10.1377/hlthaff.2011.0630 21976341

[24] The Leadership Conference on Civil and Human Rights, The Leadership Conference Education Fund, The Lawyers’ Committee for Civil Rights Under Law, the National Association for the Advancement of Colored People. Falling further behind: combatting racial discrimination in America. 2014. Available at: https://tbinternet.ohchr.org/Treaties/CERD/Shared%20Documents/USA/INT_CERD_NGO_USA_17654_E.pdf. Accessed September 3, 2020.

[25] Deer S . Native people and violent crime: gendered violence and tribal jurisdiction. Du Bois Rev. 2018;15(1):89‐106.

[26] Perry B . Impacts of disparate policing in Indian Country. Policing Soc. 2009;19(3):263‐281.

[27] Rosay AB . Violence against American Indian and Alaska Native women and men. 2010 findings from the National Intimate Partner and Sexual Violence Survey. National Institute of Justice. 2016 [cited 2019 June 20]. Available at: https://www.ncjrs.gov/pdffiles1/nij/249736.pdf.

[28] Hansen E. The forgotten minority in police shootings. CNN. 2017. Available at: https://www.cnn.com/2017/11/10/us/native-lives-matter/index.html. Accessed September 3, 2020.

[29] Kohut A , Keeter S , Doherty C , et al. Assessing the Representativeness of Public Opinion Surveys. Washington, DC: Pew Research Center. 2012. Available at: http://www.people-press.org/2012/05/15/assessing-the-representativeness-of-public-opinion-surveys/. Accessed September 3, 2020.

[30] Yaeger DS , Krosnick JA , Chang L , et al. Comparing the accuracy of RDD telephone surveys and internet surveys conducted with probability and non‐probability samples. Public Opin Q. 2011;75(4):709‐747.

[31] Keeter S , Kennedy C , Dimock M , Best J , Craighill P . Gauging the impact of growing nonresponse from a national RDD telephone survey. Public Opin Q. 2006;70(5):759‐779.

[32] Akee R . How COVID‐19 is impacting indigenous peoples in the U.S. PBS News Hour. 2020. Available at: https://www.pbs.org/newshour/nation/how-covid-19-is-impacting-indigenous-peoples-in-the-u-s. Accessed September 3, 2020.

