# Supplementary material for: The Unseen Picture: Issues with Health Care, Discrimination, Police and Safety, and Housing Experienced by Native American Populations in Rural America
Source: J Rural Health. 2020 Oct 6;38(1):180–6. doi: 10.1111/jrh.12517 (PMC9290671; doi:10.1111/jrh.12517)
Supplement: Supplementary file 1 — Supporting Information Available Online: Appendices A and B [file JRH-38-180-s001.docx]

**The Unseen Picture: Issues with Health Care, Discrimination, Police and Safety, and Housing Experienced by Native American Populations in Rural America**

**Appendix A – Question Wording (Surveys 1 and 2)^a^**

|  |  | |  |
| --- | --- | --- | --- |
| ***HEALTH CARE*** |  | |  |
| Experienced recent problems with health care access | Was there any time in the past few years when you needed health care but did not get it, or did you get health care every time you needed it in the past few years? (Survey 2, Q14) | |  |
| Experienced major problems paying for medical bills | Experienced recent problems paying for medical bills: Within the past few years, have you or anyone in your family ever had a problem paying for your medical bills or dental treatment, or not? (IF YES, ASK: Was that a major problem or a minor problem?) (Survey 2, Q30a) | |  |
| Experienced recent problems with health care quality | Was there any time in the past few years when you felt there was a problem with the quality of health care you received, or have you not had any problems with the quality of health care you received in the past few years? (Survey 2, Q16, Half Sample A) | |  |
| ***DISCRIMINATION*** | | |  |
| Experienced discrimination when trying to rent a room/apartment or buy a house ^b^ | Do you believe you have ever personally experienced discrimination because you are [AI/AN: “Native American” / Non-Hispanic White: “White”] when trying to rent a room or apartment or buy a house? (Survey 1, Q25, Half Sample B) | |  |
| Experienced discrimination in police interactions | Do you believe you have ever personally experienced discrimination because you are [AI/AN: “Native American” / Non-Hispanic White: “White”] when interacting with police? (Survey 1, Q17, Half Sample A) | |  |
| Avoided calling the police because of concerns of discrimination | Have you ever avoided calling the police or other authority figures, even when in need, out of concern that you or others in your family would be discriminated against because you or they are [AI/AN: “Native American” / Non-Hispanic White: “White”] (Survey 1, Q88a, Half Sample A) | |  |
| Experienced discrimination when going to a doctor or health clinic | Do you believe you have ever personally experienced discrimination because you are [AI/AN: “Native American” / Non-Hispanic White: “White”] when going to a doctor or health clinic? (Survey 1, Q20, Half Sample B) | |  |
| Avoided doctor or health care because of concerns of discrimination/poor treatment | Have you ever avoided going to a doctor or seeking health care for you or others in your family out of concern that you would be discriminated against or treated poorly because you or they are [AI/AN: “Native American” / Non-Hispanic White: “White”]? (Survey 1, Q62a, Half Sample B) | |  |
| ***POLICE AND SAFETY*** |  |  |  |
| Been threatened or harassed | Sum of “yes” responses to these two questions:  a) Do you believe that you or someone in your family has experienced sexual harassment because you or they are [AI/AN: “Native American” / Non-Hispanic White: “White”]? (Survey 1, Q91a, Half Sample A)  b) Do you believe that you or someone in your family has been threatened or non-sexually harassed because you or they are [AI/AN: “Native American” / Non-Hispanic White: “White”]? (Survey 1, Q91b, Half Sample A) | |  |
| Experienced violence | Do you believe that you or someone in your family has experienced violence because you or they are [AI/AN: “Native American” / Non-Hispanic White: “White”]? (Survey 1, Q91e, Half Sample A) | |  |
| Rated local community as unsafe from crime | How safe, if at all, would you say your local community is from crime? Would you say it is very safe, somewhat safe, not too safe, or not at all safe? Survey 2, Q37, Half Sample A) (Reported as safe—very/somewhat or unsafe—not too/not at all safe) | |  |
| Unfairly stopped or treated by the police | Do you believe that you or someone in your family has been unfairly stopped or treated by the police because you or they are [AI/AN: “Native American” / Non-Hispanic White: “White”]? (Survey 1, Q91c, Half Sample A) | |  |
| Unfairly treated by the courts | Do you believe that you or someone in your family has been unfairly treated by the courts because you or they are [AI/AN: “Native American” / Non-Hispanic White: “White”]? (Survey 1, Q91d, Half Sample A) | |  |
| ***HOUSING*** |  | |  |
| Experienced any housing problems | Sum of “major problem” responses to the following questions (Survey 2, Q35) Thinking now of the time you’ve lived in your current housing situation, have you ever had a problem with (INSERT ITEM)? How about (INSERT ITEM)? (Scramble items) (IF YES, ASK: And would you say that is a major problem or a minor problem?)  a. Inadequate heating or cooling  b. Mold or other environmental problems  c. Pests, bugs, or wild animals  d. The safety of your drinking water  e. Phone service  f. The sewage system  g. Electricity  h. Trash collection | |  |
| Experienced major problems paying for housing | Experienced recent problems paying for housing: Within the past few years, have you or anyone in your family ever had a problem paying for your rent or on a house payment, or not? (IF YES, ASK: Was that a major problem or a minor problem?) (Survey 2, Q30b) | |  |
| Reported homelessness is a problem in the local community | Is homelessness a problem in your local community, or not? (Survey 2, Q34, Half Sample D) | |  |

^a^ Survey 1 conducted January 26 – April 9, 2017; Survey 2 conducted January 31 – March 2, 2019; both surveys jointly designed by Harvard T.H. Chan School of Public Health, the Robert Wood Johnson Foundation, and National Public Radio. Authors’ analysis of 2 surveys of 317 Native American and 1,066 non-Hispanic White adults ages 18 and older living in the rural US (total N across both surveys, all analyses use weighted data). ^b^ Housing question only asked among respondents who have ever tried to rent a room or apartment, or to apply for a mortgage or buy a home.

| **Appendix B – Differences in Reported Experiences with Housing Problems Between Rural Native Americans and Rural Whites ^a^** | | | | |  |
| --- | --- | --- | --- | --- | --- |
|  | Rural Native Americans | Rural Whites |  |  |  |
|  | Weighted % | Weighted % | *P* value for difference |  |  |
| *HOUSING* |  |  |  |  |  |
| Experienced any major housing problems | 48% | 26% | <.001* |  |  |
| Sum of the following: |  |  |  |  |  |
| Major problems with inadequate heating or cooling | 18% | 5% | .005* |  |  |
| Major problems with mold or other environmental problems | 14% | 6% | .049* |  |  |
| Major problems with pests, bugs, or wild animals | 14% | 9% | .227 |  |  |
| Major problems with the safety of your drinking water | 18% | 7% | .018* |  |  |
| Major problems with phone service | 16% | 5% | .015* |  |  |
| Major problems with the sewage system | 9% | 5% | .299 |  |  |
| Major problems with electricity | 14% | 4% | .025* |  |  |
| Major problems with trash collection | 8% | 3% | .158 |  |  |

^a^ Authors’ analysis of 139 Native American and 892 non-Hispanic White adults living in the rural US (all analyses use weighted data). Don’t know/refused responses included in the total N. ^*^ Rural Native American adults significantly different from rural White adults at *P* < .05.
